# Improved Infraorbital Hollowing in Chinese Adults Following Hyaluronic Acid-based Filler With Lidocaine (VYC-15L) Treatment

**DOI:** 10.1093/asj/sjaf267

**Published:** 2026-01-07

**Authors:** Nanze Yu, Xiaojun Wang, Wenyu Wu, Hongyi Zhao, Zhenyu Chen, Yan Wu, Qian Tan, Ziyang Liu, Smita Chawla

## Abstract

**Background:**

Infraorbital hollowing (IOH) is characterized by a sunken appearance of the lower eyelid. The soft tissue filler VYC-15L has been demonstrated to be effective in correcting IOH in US and European populations.

**Objectives:**

The aim was to assess the safety and effectiveness of VYC-15L treatment in Chinese adults with moderate to severe IOH.

**Methods:**

In this randomized, multicenter, 12-month study, the primary endpoint was the proportion of ≥1-grade improvements on the Allergan Infraorbital Hollowing Scale (AIHS) according to the evaluating investigator (EI) at Month 3. Secondary endpoints included EI-based and participant-based assessments with the Global Aesthetic Improvement Scale (GAIS) and FACE-Q responses evaluated at Month 3. Safety was monitored throughout.

**Results:**

The primary endpoint was met with a 94.6% AIHS responder rate in the VYC-15L group vs 0.0% in the delayed-treatment control (DTC) group (*P* < .0001). Significant GAIS improvements in VYC-15L vs DTC participants were seen in EI-assessed (99.1% vs 0.0%) and participant-assessed (95.6% vs 5.7%) responder rates at Month 3 (*P* < .0001). The mean change in FACE-Q scores was greater in the VYC-15L group than the DTC group at Month 3 (*P* < .0001). Improvements from VYC-15L treatment were seen across all effectiveness measures through Month 12. Mean pain scores were 2.9. Most injection site reactions were mild and resolved within 14 days. Forty-six participants (28.8%) experienced treatment-emergent adverse events (TEAEs), including treatment-related TEAEs (1.7%), which were blepharospasm (0.8%) and injection site bruising (0.8%). No serious TEAEs were reported.

**Conclusions:**

VYC-15L was effective and well tolerated for IOH correction in Chinese adults over up to 1 year.

**Level of Evidence: 1 (Therapeutic):**

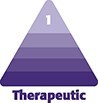

Infraorbital hollowing (IOH) is a common aesthetic concern characterized by a sunken appearance in the lower eyelid region and may contribute to a tired and aging facial appearance.^1^ Age-related changes underlying IOH include remodeling of bones, fat pads, ligaments, and musculature.^2^ Several treatment options are available, including subdermal filling, autologous fat transfer, topical agents, lower eyelid blepharoplasty, and laser resurfacing.^2^ Hyaluronic acid (HA)-based soft tissue fillers have become a popular minimally invasive option for addressing IOH, given the short recovery time and high patient satisfaction.^3-5^

Although many studies of aesthetic treatments include mostly White patients, there is a growing interest in aesthetic treatments among Asian populations.^6^ There are notable anatomical differences in Asian faces when compared with White faces, which are important to consider for determining patient treatment plans and outcomes.^6^ This includes Asian faces often having a thicker dermis and a greater amount of collagen than White faces.^7^ Additionally, the infraorbital cheek region in Asian faces contains more fat tissue and weaker skeletal support than White faces, possibly leading to greater facial sagging.^8^ Given these differences, it is critical to assess IOH treatment outcomes in an Asian patient population.

VYC-15L (Juvéderm Volbella with Lidocaine; Allergan Aesthetics, an AbbVie company, Irvine, CA) is an HA soft tissue filler containing 15 mg/mL HA with 0.3% weight by weight (w/w) lidocaine, which is added to reduce injection-related pain and provide consistency in anesthetic dosing.^9^ The proprietary mix of low-molecular weight and high-molecular weight HA as well as the low elastic modulus (G′ ≈ 160 Pa) of VYC-15L means this product is designed to be well-suited for treatment of areas such as the perioral area, lips, and tear troughs due to its moldability (ie, spreading, modeling, shaping) and ease of flow during injection.^10^ VYC-15L has been approved by the United States, Canada, Australia, and the European Union for various aesthetic indications, including lip augmentation and IOH treatment.^11,12^ It also is approved for correction of lip structural defects treatment in China.^13^ Here, we evaluated the safety and effectiveness of VYC-15L for correcting IOH in adult Chinese participants.

## METHODS

### Study Design

**Figure 1.**
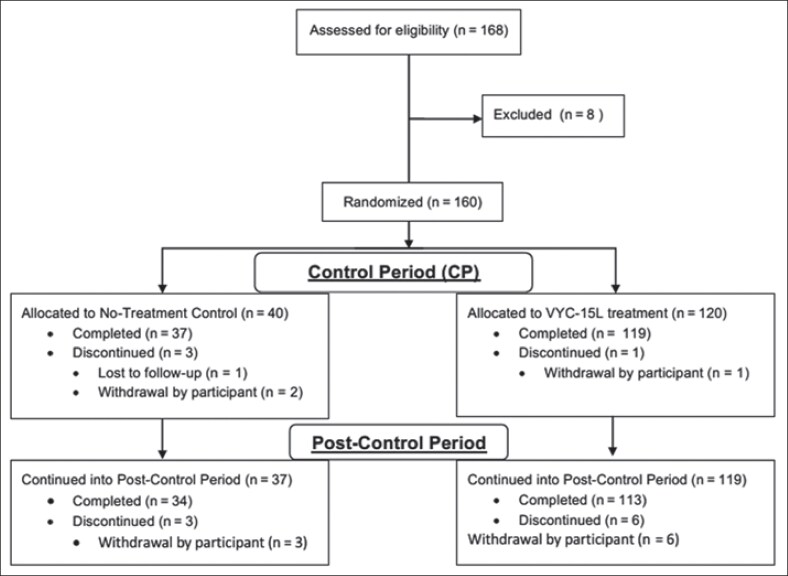
CONSORT flow diagram.

This was a prospective, multicenter, evaluator-blinded, randomized, parallel-group, no-treatment controlled trial conducted at 6 sites in China from October 2021 to November 2023. Before the study was initiated, with an interactive web response system at each study site, participants were randomized 3:1 to receive either VYC-15L treatment or to serve as no-treatment controls (Figure 1). Following randomization, participants in the VYC-15L treatment group received an initial and optional touch-up treatment at Month 1 if needed to achieve optimal correction. No-treatment control participants were followed for 3 months postrandomization, during which they received no treatment. After the initial 3-month follow-up period, no-treatment control participants were offered the option to receive the VYC-15L treatment and were considered part of the delayed-treatment control group (DTC). Both initial and delayed VYC-15L treatment groups had follow-up visits, which occurred at Day 7 and at Months 1, 3, 6, 9, and 12 posttreatment. All participants remained in the study for up to 14 months.

The study adhered to the Independent Ethics Committee (IEC), was conducted in compliance with ICH guidelines and the Declaration of Helsinki and was registered at clinicaltrials.gov (NCT# 05088980). All study centers obtained IEC approval for all relevant study documentation, including the protocol and informed consent forms.

### Participant Selection and Eligibility Requirements

Chinese adult participants with moderate or severe infraorbital hollowing, defined as a Grade 2 or 3, respectively, on the Allergan Infraorbital Hollowing Scale (AIHS) for each eye participated in the study. The AIHS is a validated 5-point ordinal scale administered to assess the severity of IOH, ranging from 0 (none) to 4 (extreme). Both eyes had to be rated a 2 or 3 on the AIHS scale according to the evaluating investigator (EI), however they did not need to have the same severity score. Key exclusion criteria included previous facial surgeries or treatments that could potentially interfere with study assessments, such as permanent facial implants or botulinum toxin injections within 9 months before study entry; residual deficiencies, deformities, or scarring in the periorbital or cheek areas; atrophic skin in the infraorbital region; hyperpigmentation in the infraorbital area; or large lower fat pads that would mask improvement. Female participants who were pregnant, nursing, or planning a pregnancy also were excluded.

### Treatment Plan and Injection Pattern

Before treatment, the treatment area was thoroughly cleaned, prepared with alcohol or antiseptic, and topical anesthesia or ice was applied at the discretion of the treating investigator (TI). Following aspiration, VYC-15L was slowly injected directly into the supraperiosteal plane of the infraorbital area with a 32-gauge 1/2-inch needle with up to 3 boluses utilizing a depot injection technique. Injectors were advised to insert the needle below the orbital rim perpendicular to the skin surface and advance in an anterior to posterior direction until the periosteum was contacted. The injection area could extend approximately 2 cm below the inferior orbital rim to provide optimal results. The maximum injection volume allowed was up to 2.2 mL per side for both initial and optional touch-up treatments.

### Effectiveness Endpoint

The primary effectiveness endpoint was responder status, defined as a 1-grade improvement on the AIHS scale, based on the EI's live assessment at Month 3. AIHS responder status also was assessed over time, with live EI assessments of AIHS ratings at Months 1, 3, 6, 9, and 12. Similarly, the responder status (improved or much improved) for participant and EI assessments of improvements on the Global Aesthetic Improvement Scale (GAIS), ranging from −2 = much worse to 2 = much improved, at Months 1, 3, 6, 9, and 12 were evaluated. The FACE-Q Appraisal of Lower Eyelids questionnaire was administered to participants to rate participant satisfaction at Months 1, 3, 6, 9, and 12. Last, changes in infraorbital volume as assessed by 3-dimensional (3D) image volumetric analysis at Months 1, 3, 6, 9, and 12 also were captured.

### Injection Ease and Product Moldability

TIs rated injections for the ease of injection and product moldability on 11-point scales, ranging from 0 = difficult to 10 = easy and 0 = stiff to 10 = moldable, respectively.

### Safety

Procedural pain was rated with an 11-point scale ranging from 0 (no pain) to 10 (worst pain imaginable) immediately following both initial and touch-up treatments, as performed in previous VYC-15L studies.^5,14,15^ Participants documented the frequency, severity, and duration of injection site reactions (ISRs) in an e-dairy for 30 days following each treatment session. At each follow-up visit, TIs reviewed participant diaries to determine whether the ISR qualified as an adverse event (AE), and the type of intervention required to resolve the reaction. AEs were monitored through the study. Vital signs and laboratory evaluations were measured throughout the study. The presence of a Tyndall effect was assessed by the EI at Month 1.

### Statistical Methods

The primary effectiveness endpoint was analyzed with a 2-sided Fisher’s exact test to compare the AIHS responder rate between treatment and control groups at Month 3. Safety outcomes were summarized through descriptive statistics. Missing data for primary effectiveness were addressed with multiple imputations. The significance level for statistical tests was set at *P* = .05, and 95% confidence intervals (CIs) were reported when applicable.

## RESULTS

### Participant Demographics and Baseline Characteristics

Of the 160 randomized participants, 156 participants (97.5%) completed the Month 3 control period and 147 (91.9%) completed the study. The most common reason for study discontinuation was withdrawal by participant (1.9%) (Figure 1). During the control period, the VYC-15L treatment group included 120 participants and the DTC group included 40 participants. From the initial VYC-15L treatment population (*n* = 120), 49 (40.8%) received a touch-up treatment under 1 or both eyes at Month 1. Following the control period, 37 DTC participants continued in the study and all (100.0%) received optional delayed-treatment. Of the 37 DTC participants, 15 (40.5%) participants received an optional touch-up treatment at Month 4. Participants were a mean age of 33.8 years (range, 22-59 years), and a majority self-identified as female (females, *n*  = 152: 95.0%; males, *n*  = 8: 5.0%) and Chinese (100.0%). Baseline IOH severity ratings were similar between the DTC group (right infraorbital area: moderate, 50.0%; severe, 50.0%; left infraorbital area: moderate, 42.5%, severe, 57.5%) and the VYC-15L treatment group (Table 1).

**Table 1.** Participant Demographics and Baseline Characteristics

	Delayed-treatment control (*n* = 40)	VYC-15L (*n* = 120)	Total (*n* = 160)
Mean age, years (SD)	34.1 (8.06)	33.7 (7.49)	33.8 (7.61)
Sex			
Female, *n* (%)	36 (90.0)	116 (96.7)	152 (95.0)
Male, *n* (%)	4 (10.0)	4 (3.3)	8 (5.0)
Race			
Chinese, *n* (%)	40 (100.0)	120 (100.0)	160 (100.0)
Allergan Infraorbital Hollowing Scale score, *n* (%)			
Right infraorbital area			
Moderate (Grade 2)	20 (50.0)	58 (48.3)	78 (48.8)
Severe (Grade 3)	20 (50.0)	62 (51.7)	82 (51.3)
Left infraorbital area			
Moderate (Grade 2)	17 (42.5)	59 (49.2)	76 (47.5)
Severe (Grade 3)	23 (57.5)	61 (50.8)	84 (52.5)

SD, standard deviation.

### Treatment Administration

The total combined median volume for initial and touch-up treatments was 2.8 mL, with a median of 2.0 mL for initial treatment and a median of 1.0 mL for touch-up treatment for both eyes (Table 2). All injections were administered as boluses to the submuscular/supraperiosteal plane in the left and right infraorbital areas. The investigators gave high ratings for ease of VYC-15L injection and moldability, as the majority of participants received a score of ≥9, TI-rated, for both measures after initial (ease, 68.4%; moldability, 74.2%) and touch-up (ease, 75.5%; moldability, 75.5%) treatment.

**Table 2.** Key Injection Parameters

	Initial treatment		Delayed treatment	
	Initial (*n* = 120)	Touch-up (*n* = 49)	Initial (*n* = 37)	Touch-up (*n* = 15)
Median volume, mL (min, max)	2.00 (0.45, 4.00)	1.00 (0.05, 4.00)	2.00 (0.60, 4.00)	0.90 (0.10, 2.00)
Injection instrument, *n* (%)				
32-gauge 1/2-inch needle	120 (100.0)	49 (100.0)	37 (100.0)	15 (100.0)
Pretreatment anesthesia, *n* (%)				
Ice	6 (5.0)	4 (8.2)	1 (2.7)	1 (6.7)
Topical anesthesia	34 (28.3)	11 (22.4)	10 (27.0)	3 (20.0)
Local injectable anesthesia	0 (0.0)	0 (0.0)	0 (0.0)	0 (0.0)
Planes of injection, *n* (%)				
Submuscular/supraperiosteal	120 (100.0)	49 (100.0)	37 (100.0)	15 (100.0)
Product characteristics scores, *n* (% of ≥9)				
Ease of injection	82 (68.4)	37 (75.5)	31 (83.8)	9 (60.0)
Moldability	89 (74.2)	37 (75.5)	31 (83.8)	10 (66.7)

### Effectiveness

**Figure 2.**
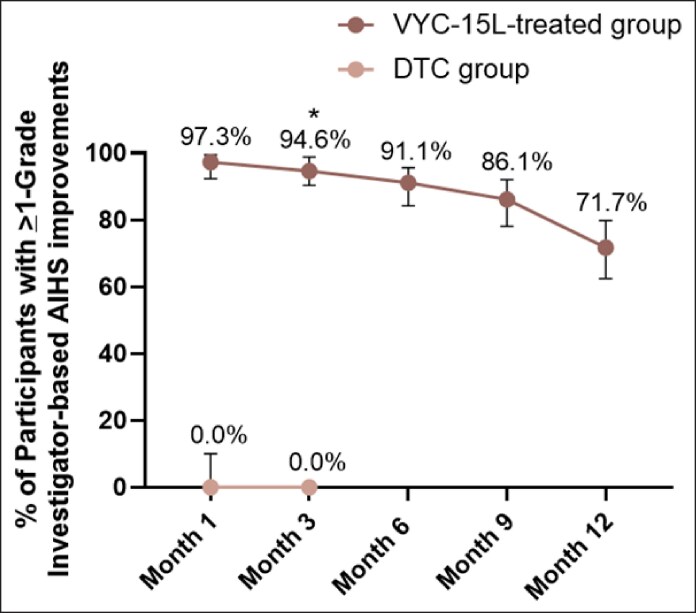
Proportion of participants with ≥1-grade investigator-based Allergan Infraorbital Hollow Scale (AIHS) improvements throughout the 12-month study. DTC, delayed-treatment control.

The primary effectiveness endpoint of achieving ≥1-grade improvement on the AIHS at Month 3 showed a significantly higher responder rate in the VYC-15L group compared with the DTC group (difference, 94.6%; CI, 90.39-98.83; *P* < .0001; Figure 2). Improvements in AIHS scores associated with VYC-15L treatment continued throughout the study, with a 71.7% responder rate observed at Month 12.

**Figure 3.**
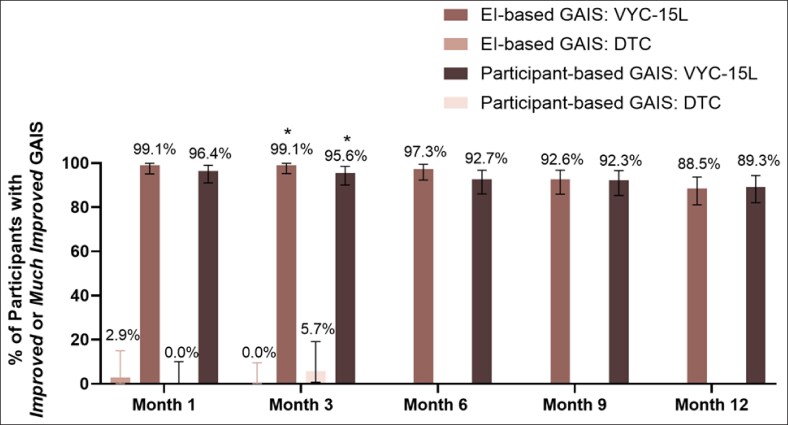
Percentage of participants rated by investigators or participants as improved or much improved on the GAIS throughout the 12-month study. DTC, delayed-treatment control; EI, evaluating investigator; GAIS, Global Aesthetic Improvement Scale.

**Figure 4.**
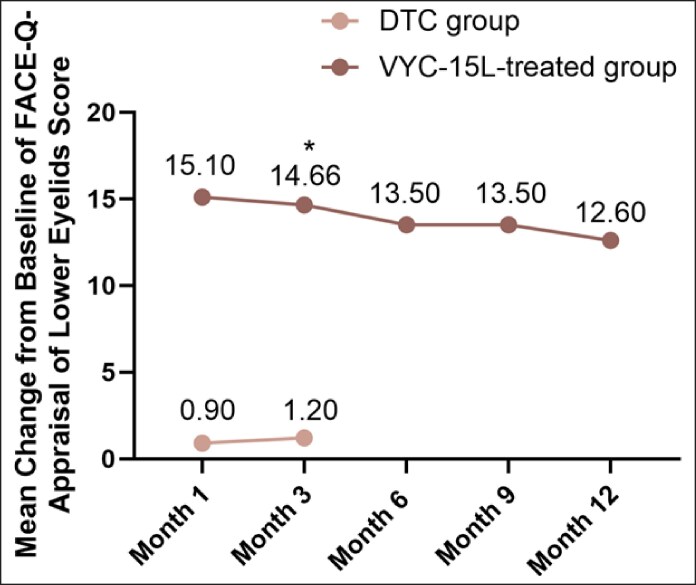
Mean change from baseline of FACE-Q appraisal of lower eyelids score in participants throughout the study. DTC, delayed-treatment control.

Secondary endpoints further supported the effectiveness of VYC-15L treatment, with 99.1% of EI-assessed and 95.6% of participant-assessed GAIS responses indicating improvement at Month 3 in VYC-15L group (*P* < .0001 for both compared to DTC; Figure 3). High improvements according to both participants and the EI following VYC-15L treatment continued through Month 12. Participants treated with VYC-15L also reported significantly higher mean changes from baseline at Month 3 in FACE-Q Appraisal of Lower Eyelids scores than DTC participants (12.6 vs 1.2; *P* < .0001; Figure 4). For instance, 62.7% of VYC-15L–treated participants reported being “moderately bothered” or “extremely bothered” at baseline with how old the area under their eyes make them look, which decreased to 19.3% at Month 3 of the study, representing improvement of the IOH. Mean changes from baseline of FACE-Q scores remained consistently high throughout the study.

**Figure 5.**
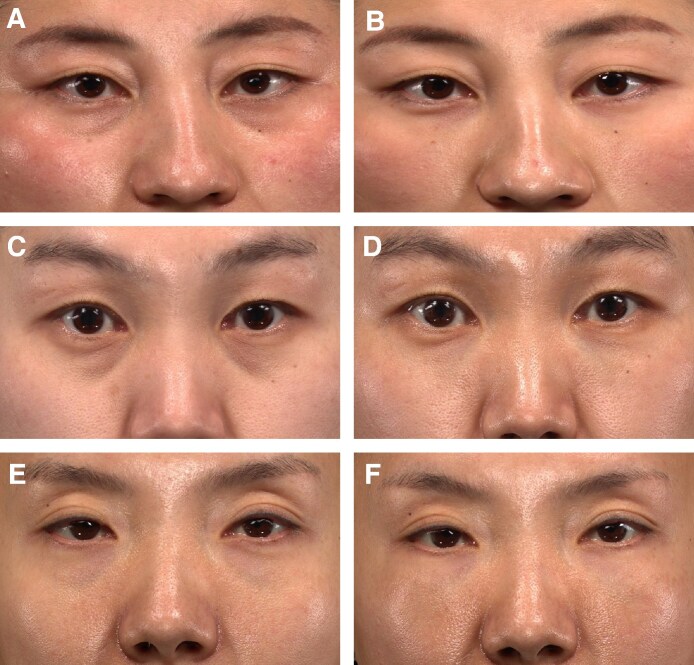
Representative photographs of AIHS improvements in participants from baseline to Month 3. (A) Pretreatment photo of a 24-year-old female participant who achieved a 3-grade AIHS improvement following treatment with VYC-15L, with baseline AIHS score of 3; (B) posttreatment photo at Month 3 with AIHS score of 0 following initial treatment volume of 1.0 mL (right side) and 1.0 mL (left side), and an additional 0.5 mL (left side) at the Month 1 touch-up. (C) Pretreatment photo of a 31-year-old female participant who achieved a 2-grade AIHS improvement following treatment with VYC-15L, with baseline AIHS score of 3; (D) posttreatment photo at Month 3 with AIHS score of 1 following initial treatment volume of 1.8 mL (right side) and 1.85 mL (left side). (E) Pretreatment photo of a 37-year-old female participant who achieved a 3-grade AIHS improvement following treatment with VYC-15L, with baseline AIHS score of 3; (F) posttreatment photo at Month 3 with AIHS score of 0 following initial treatment volume of 2.0 mL (right side) and 2.0 mL (left side). AIHS, Allergan Infraorbital Hollow Scale.

Three-dimensional imaging assessments showed a mean change from baseline volume of 1.2 mL for each eye in VYC-15L treatment participants and −0.1 mL for each eye in DTC participants at Month 3. Participants who initially received VYC-15L treatment continued to have a mean change from baseline volume of 1.2 mL at Months 1, 6, and 12. Representative photographs of participants who achieved a 2- or 3-grade improvement on AIHS with VYC-15L treatment are shown in Figure 5.

### Safety

#### Procedural Pain

Participants reported low mean procedural pain scores immediately following initial (2.9) and touch-up (2.6) treatment. Mean scores were slightly higher for delayed-treatment participants receiving optional initial (3.2) and touch-up treatment (3.5).

#### Injection Site Reactions (ISRs)

**Figure 6.**
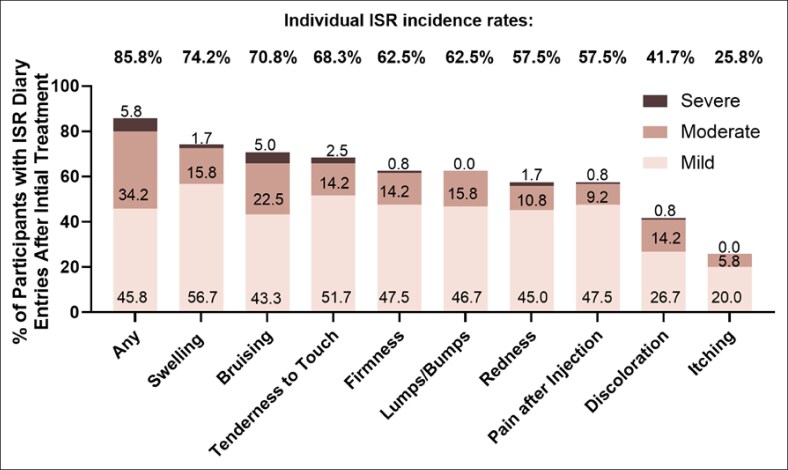
Incidence of injection-site responses (ISRs) after initial treatment.

At least 1 ISR following initial treatment was reported by 85.8% of the initially treated participants (Figure 6). The most frequent ISRs included swelling (74.2%), bruising (70.8%), and tenderness to touch (68.3%). Similar trends were observed for touch-up treatments, with 69.4% of participants reporting ISRs. The mean ISR duration of the initial VYC-15L treated population was 14.5 days after initial and 13.9 days after touch-ups treatments, with most being mild to moderate in severity. For the delayed-treatment participants, ISR occurred in 81.1% of participants and were mild or moderate in severity, with the most frequently reported ISRs being swelling (81.1%), lump/bumps (73.0%), and bruising (73.0%). The mean duration of ISRs in the delayed-treatment population was 20.5 days after initial treatment and 16.6 days after touch-up treatment.

#### Tyndall Effect

At Month 1 of the treatment period, bilateral presentations of the Tyndall effect were seen in 23 (19.2%) and 24 (20.0%) initially treated participants for the right and left infraorbital areas, respectively. Similar rates of Tyndall effect were seen in the delayed-treatment group.

#### Adverse Events

Within the initial treatment period, 75.8% of initially treated participants and 78.4% of the delayed-treatment participants reported at least 1 treatment-emergent adverse event (TEAE) (Table 3). With the exception of COVID-19 infection, the most common TEAEs reported by at least 3 initially treated participants were acne, eczema, menstrual disorder (female participants only), pharyngitis, spinal osteoarthritis, and upper respiratory tract infection. In the delayed-treatment participants, with the exception of COVID-19 infection, the most common TEAEs reported by at least 2 participants were upper respiratory tract infection and eczema. No TEAEs related to vision function were reported. All TEAEs were mild or moderate in severity.

**Table 3.** Summary of Adverse Events and Treatment-Emergent Adverse Events

	Initial treatment (*n* = 120)	Delayed treatment (*n* = 37)	
	Initial + touch-up (*n* = 120)	Initial (*n* = 37)	Touch-up (*n* = 15)
All TEAEs, *n* (%)	91 (75.8)	29 (78.4)	
Treatment-related TEAEs	2 (1.7)	0 (0.0)	
At injection site	1 (0.8)	0 (0.0)	
Not at injection site	1 (0.8)	0 (0.0)	
All TESAEs, *n* (%)	5 (4.2)	2 (5.4)	
Treatment-related	0 (0.0)	0 (0.0)	
Discontinued due to TEAE, *n* (%)	0 (0.0)	0 (0.0)	
Deaths	0 (0.0)	0 (0.0)	

TEAE, treatment-emergent adverse event; TESAE, treatment-emergent serious adverse event.

Treatment-related TEAEs were seen in 2 (1.7%) of the initially treated participants with the most common being blepharospasm (0.8%) and injection site bruising (0.8%). The blepharospasm stabilized by the end of the study and the 1 reported injection site bruising event resolved within 7 days. No treatment-related TEAEs were noted in the delayed-treatment population. No treatment-related serious adverse events (SAEs) or deaths were reported throughout the study.

## DISCUSSION

Asian patients are increasingly interested in injectables and minimally invasive procedures, however many current clinical studies enroll mostly White participants to assess treatment outcomes.^16^ In this study, VYC-15L treatment in Chinese participants led to clinically meaningful improvements in IOH severity through Month 12. The primary effectiveness endpoint was met, with the VYC-15L treatment group achieving a statistically significant AIHS responder rate at Month 3 compared with the DTC group. The majority of participants in the VYC-15L treatment group achieved a ≥ 1-grade AIHS improvement through 12 months. AIHS responder rates declined over time, from 94.6% at Month 3, the primary endpoint, to 71.7% at Month 12, the end of the study. These long-term improvements are consistently seen across other VYC-15L studies in the IOH area, ranging from 71.7% to 73.4% at Month 12 posttreatment.^5,11,16^ Similarly, secondary effectiveness endpoints were achieved, with GAIS responder rates evaluated by both investigator and participants in addition to participant satisfaction rates based on FACE-Q significantly higher in VYC-15L–treated participants than the DTC at Month 3. These improvements continued through Month 12. VYC-15L also was given high ratings by investigators for the ease of injection and moldability in the infraorbital area. The mean change in volume for each eye, assessed by 3D imaging, was consistent with AIHS scores showing improvements until 1 year following VYC-15L treatment.

The injection technique in this study was similar to that in 2 other VYC-15L studies, performed in the United States and the EU. However, all investigators in this study utilized 32-gauge 1/2-inch needles to inject VYC-15L as boluses into the submuscular/supraperiosteal plane in the infraorbital areas, higher volumes than those in previous VYC-15L studies treating IOH.^11,12^ Although both cannula and needles have a similar safety profile and efficacy, needles may be preferred for making fine contours with injections into the subdermal area above the orbicularis oculi muscle.^2,12,17^ In addition, the average injected volumes were slightly higher than previous studies that had White participants, which may be due to the anatomical differences in Asian faces vs White faces.^16,18^ This includes Asian faces typically presenting with a flatter midface, which may accentuate the appearance of IOH.^19,20^ Additionally, Chinese individuals often have a receding lower bony orbital rim, an area surrounded by thin skin.^21^ This unique facial characteristic may lead to slightly larger volumes for IOH correction in Chinese patients.

This study showed that injection of VYC-15L into the infraorbital area was well tolerated by Chinese participants; most ISRs were mild to moderate in severity and resolved within 14 to 30 days. Additionally, only 2 treatment-related TEAEs were reported, blepharospasm and injection site bruising, which were both mild in severity. This safety profiles aligns with previous clinical studies that enrolled White participants for IOH treatment, which showed only mild TEAEs.^5,11^ A similar safety profile was seen for VYC-15L in lip treatment of Chinese participants.^13^ This demonstrates the cumulative safety profile of the VYC-15L for facial aesthetic treatments. Aside from 1 reported case of blepharospasm, which stabilized at the end of the study and required no medical intervention, no adverse events related to blindness occurred from VYC-15L treatment. Blindness can occur from vascular occlusion, due to the migration of a filler embolus. Therefore, it is important for injectors to have a deep understanding of facial anatomy to avoid the infraorbital artery and its numerous arterial branches and to ensure proper patient selection for treatment.^2^ Additional treatment considerations include aspirating before injection and injecting VYC-15L slowly to prevent adverse events.^22^ The reported ISRs were seen in other VYC-15L studies, including swelling, bruising, and tenderness.^5^ This study also was initiated in 2021 and continued through 2023, which accounts for more than half of participants experiencing COVID-19 in both the initially treated and delayed-treatment VYC-15L groups. The transient, mild, injection site bruising seen in this study also is a typical treatment-related TEAE associated with injection of soft tissue fillers.^5,11,13^ The inclusion of 0.3% lidocaine in VYC-15L is intended to reduce pain during the injection.^9^ Consistent with this, the low scores of procedural pain reported by participants suggest that the lidocaine in the product, along with proper treatment administration, may minimize injection pain in the infraorbital area.

This study further illustrates that IOH improvements from VYC-15L treatment are seen globally, as findings from clinical studies conducted in Europe, the United States, and now China have reported similarly consistent IOH improvements following VYC-15L treatment.^5,11,12^ With no other HA-based soft tissue filler product approved to treat IOH in China, these results are particularly important. To achieve optimal aesthetic outcomes, it is critical to tailor treatment to the individual and consider patient goals. This includes cultural differences of aesthetic ideals, which are known to differ between Asian and Western faces. Additionally, treatment of the IOH is complex given the number of baseline anatomical differences and age-related remodeling that can occur in Asian faces and differ in White faces.^16,19^ The high participant satisfaction throughout this study suggests that VYC-15L produces a desirable aesthetic result for Chinese patients seeking to improve infraorbital hollowing. Additionally, the high ratings of ease and moldability by investigators after both initial and touch-up injections may indicate the ability of VYC-15L to address injection unevenness or overcorrection with light massage.^23^ These results, in addition to previous studies, demonstrate that VYC-15L has the appropriate characteristics for the treatment of IOH.^5,11,12^

One limitation of the study is that treatment of the IOH was isolated to the infraorbital area to determine the safety and efficacy of VYC-15L for IOH treatment. However, because the infraorbital region is anatomically complex and may present unique treatment challenges, clinicians usually begin by restoring volume in the adjacent midface and cheek areas. Once these neighboring regions are corrected, the infraorbital area is targeted to achieve comprehensive and holistic IOH treatment.^19^ Treating volume loss in the midface area first may lead to more efficient product use in the IOH and may improve overall IOH appearance.^2^ Another limitation of this study is that the majority of the participants were female, and therefore we cannot generalize these results to the male population. Another study design limitation is a no-treatment control in the control period, as it may not properly address placebo effects or participant expectancy biases in the VYC-15L treatment group. Additionally, having the control group become a delayed-treatment group following the 3-month control period may not entirely account for participants' expectations. However, a control injection can be easily distinguished from a soft tissue filler injection by both the injector and participant, and therefore does not provide any additional blinding over a no-treatment control. Also, a control injection exposes participants to the pain associated with facial injections and puts them at risk for infection or ISRs with no clinical benefit. Last, although there was only 1 injection method for VYC-15L treatment in this study, other injection methods, such as a cannula, are often employed for soft tissue filler treatment.^2,16^ Tool selection for injections is critically important and therefore should be based on both the injector's preference and considerations for patient treatment outcomes.

## CONCLUSIONS

VYC-15L was effective in improving infraorbital hollowing in Chinese participants through up to 1 year, with clinically meaningful AIHS responder rates. Additionally, VYC-15L has an acceptable safety profile for treatment of IOH, as seen by all TEAEs being mild or moderate in severity, with no new safety concerns identified in Chinese participants.
